# Delineation of six species of the primitive algal genus *Glaucocystis* based on *in situ* ultrastructural characteristics

**DOI:** 10.1038/srep29209

**Published:** 2016-07-07

**Authors:** Toshiyuki Takahashi, Tomoki Nishida, Akihiro Tuji, Chieko Saito, Ryo Matsuzaki, Mayuko Sato, Kiminori Toyooka, Hidehiro Yasuda, Hisayoshi Nozaki

**Affiliations:** 1 Department of Biological Sciences, Graduate School of Science, University of Tokyo, 7-3-1 Hongo, Bunkyo-ku, Tokyo, 113-0033, Japan; 2 Research Center for Ultra-High Voltage Electron Microscopy, Osaka University, 7-1 Mihogaoka, Ibaraki, Osaka, 567-0047, Japan; 3 Department of Botany, National Science Museum, 4-1-1 Amakubo, Tsukuba, Ibaraki, 305-0005, Japan; 4 RIKEN Center for Sustainable Resource Science, 1-7-22 Suehiro-cho, Tsurumi-ku, Yokohama, Kanagawa, 230-0045, Japan

## Abstract

The field of microbiology was established in the 17^th^ century upon the discovery of microorganisms by Antonie van Leeuwenhoek using a single-lens microscope. Now, the detailed ultrastructures of microorganisms can be elucidated *in situ* using three-dimensional electron microscopy. Since the availability of electron microscopy, the taxonomy of microscopic organisms has entered a new era. Here, we established a new taxonomic system of the primitive algal genus *Glaucocystis* (Glaucophyta) using a new-generation electron microscopic methodology: ultra-high-voltage electron microscopy (UHVEM) and field-emission scanning electron microscopy (FE-SEM). Various globally distributed *Glaucocystis* strains were delineated into six species, based on differences in *in situ* ultrastructural features of the protoplast periphery under UHVEM tomography and in the mother cell wall by FE-SEM, as well as differences in the light microscopic characteristics and molecular phylogenetic results. The present work on *Glaucocystis* provides a model case of new-generation taxonomy.

Scientists were unaware of the existence of microorganisms until their discovery in 1674 by Antonie van Leeuwenhoek using a single-lens microscope, thereby establishing the field of microbiology^1^,^2^. Electron microscopy (EM), developed in the 20^th^ century, has also contributed much to our understanding of microorganism ultrastructural characteristics^3^,^4^,^5^. However, morphological delineation of species in unicellular or colonial organisms has been limited compared with that in macroorganisms, especially in terms of their three-dimensional (3D) characteristics^6^,^7^.

Glaucophytes constitute one major lineage of such microorganisms. They are rare freshwater algae retaining the most ancestral features of primary photosynthetic eukaryotes or Archaeplastida^7^, which also include red algae and Chloroplastida (green algae and land plants^7^). Thus, glaucophyte algae represent an evolutionarily important group within Archaeplastida. However, species concepts of glaucophytes have fallen behind those in other archaeplastidal lineages, because the ability to determine morphological differences by light microscopy (LM) and conventional EM is limited^8^,^9^,^10^ (see Supplementary Note). Schnepf *et al.*^8^ observed three strains of the glaucophyte genus *Glaucocystis* (SAG 229-1, SAG 229-2 and SAG 229-3) by ultrathin-section transmission electron microscopy (TEM) and reported no ultrastructural differences among them. However, since their observations, no comparative morphological studies using multiple strains of *Glaucocystis* were performed until recently (see below).

Although TEM has sufficiently high resolution to elucidate precise characteristics, even in 10-μm-scale microalgae, conventional TEM can reveal only limited parts of cells, *locally*, using ultrathin samples into which the electron beam are transmitted^3^,^4^,^5^. On the other hand, scanning electron microscopy (SEM) can reveal the characteristics of the entire cell surface, *globally*, but conventional SEM does not have sufficiently high resolution to observe ultrastructures in detail^10^,^11^. Recently, two types of new-generation EM, ultra-high-resolution (UHR) field-emission (FE)-SEM and ultra-high-voltage electron microscopy (UHVEM), have introduced a new paradigm in the field of biology^10^,^11^,^12^,^13^,^14^. Despite this, in some recent studies of microalgal and protozoan taxonomy, the utility of molecular approaches continues to be overemphasised^15^,^16^,^17^,^18^.

UHR FE-SEM enables ultrafine observations of the entire cell surface even at low accelerating voltages (LV); it also allows *in situ* surface ultrastructures in numerous cells to be observed all at once^10^,^11^,^12^. Our recent study using LV FE-SEM unveiled species diversity within the flagellate glaucophyte genus *Cyanophora*, identifying three new species^10^. However, LV FE-SEM cannot be applied to examine a protoplast enclosed by a cell wall, as in the coccoid glaucophyte genus *Glaucocystis*^19^,^20^. For this, UHVEM, which enables *in situ* 3D ultrastructural observation by thick-section tomography using an ultra-high accelerating voltage, can be used^5^. Recently, 3D UHVEM tomography revealed morphological diversity in terms of the 3D ultrastructure of the protoplast periphery using three divergent strains of *Glaucocystis*^13^,^14^. Thus, undescribed species of this genus are expected to be delineated morphologically among strains distributed across the globe, based on new-generation EM characteristics.

Here, we aimed to delineate morphologically and phylogenetically different *Glaucocystis* species based on the combination of several types of microscopy, including 3D UHVEM tomography and LV FE-SEM, combined with molecular phylogenetic results, from 10 globally distributed strains labelled *G. nostochinearum* Itzigs. *ex* Rabenh. (1866)^21^,^22^ and three newly established strains of *Glaucocystis* (Supplementary Table 1). A new taxonomic system of *Glaucocystis* species delineated using new-generation EM is described in this report (Table 1).

## Results

### Light microscopy

**Figure 1:**
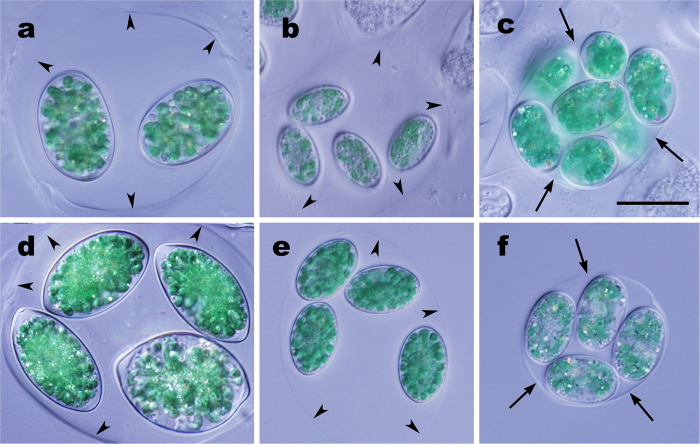
Differential interference contrast microscopy of colonies of six *Glaucocystis* species. Shown at the same magnification. Scale bar, 20 μm. Note that each colony is enclosed by mother cell wall (arrows) tightly (**c,f**) or arranged in a less crowded way within an extended mother cell wall (arrowheads) (**a,b,d,e**). (**a**) *G. geitleri* E.G.Pringsh. *ex* Tos.Takah. & Nozaki sp. nov. strain SAG 229-1. (**b**) *G. nostochinearum* Itzigs. *ex* Rabenh. strain SAG 16.98. (**c**) *G. incrassata* (Lemmerm.) Tos.Takah. & Nozaki stat. nov. strain SAG 229-2. (**d**) *G. oocystiformis* Prescott strain 126. (**e**) *G. miyajii* Tos.Takah. & Nozaki sp. nov. strain Thu10. (**f**) *G. bhattacharyae* Tos.Takah. & Nozaki sp. nov. strain 118.

Using LM on the 13 strains (Supplementary Table 1), two *Glaucocystis* species were identified based on the traditional taxonomic system^19^,^23^,^24^ (Supplementary Table 2): *G. nostochinearum* and *G. oocystiformis* Prescott (1944)^23^ (see Supplementary Note). Moreover, we found differences that could contribute to species delineation within *G. nostochinearum* in our new taxonomic system (Table 1; Fig. 1; Supplementary Figs 1,2; Supplementary Note).

### Field-emission scanning electron microscopy

The cell wall of *Glaucocystis* is composed of cellulose filaments and has the highest cellulose I_α_ crystallite content of all organisms^25^,^26^,^27^,^28^. The cellulose filament structure derived from this alga was previously examined by TEM and several types of spectroscopy^25^,^26^,^27^,^28^,^29^,^30^. However, FE-SEM was not yet used to reveal the *in situ* ultrastructural surface of the *Glaucocystis* colony or mother cell wall.

**Figure 2:**
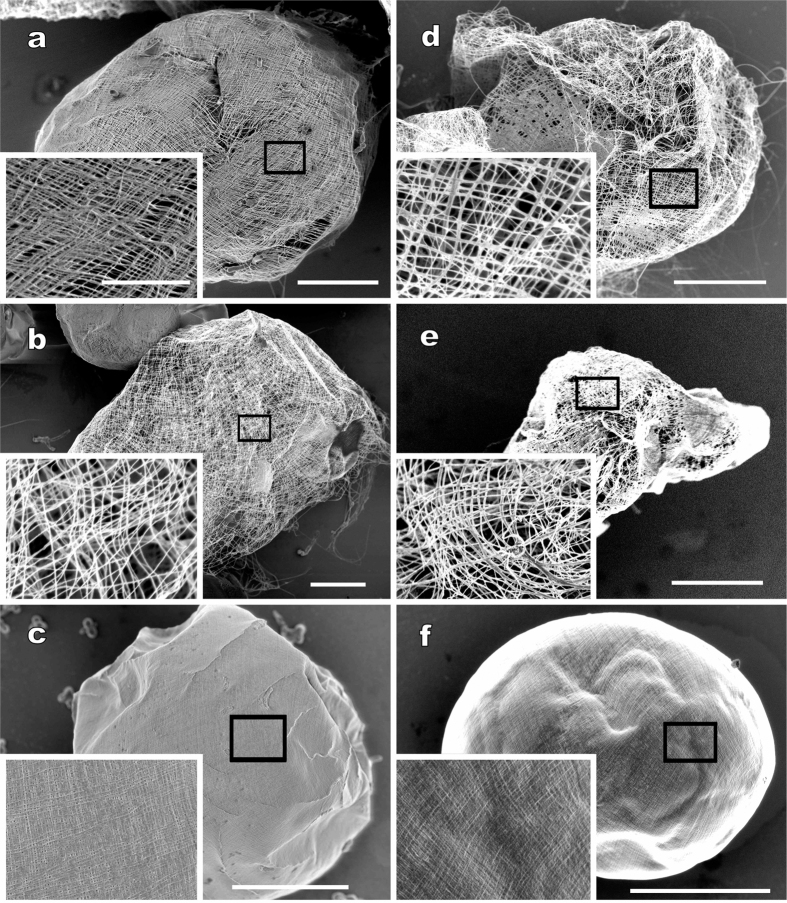
Field-emission scanning electron microscopy of colonies of six *Glaucocystis* species. Insets show higher magnification image of the mother cell wall surface (boxed area) at the same magnification. Scale bar, 10 μm and 2 μm (insets). Note that each colony is enclosed by a mother cell wall, showing gauze fabric-like fibrils globally (**a,b,d,e**) or tightly arranged fibrils (**c,f** ). (**a**) *G. geitleri* E.G.Pringsh. *ex* Tos.Takah. sp. nov. strain SAG 229-1. (**b**) *G. nostochinearum* Itzigs. *ex* Rabenh. strain SAG 16.98. (**c**) *G. incrassata* (Lemmerm.) Tos.Takah. stat. nov. strain SAG 229-2. (**d**) *G. oocystiformis* Prescott strain 126. (**e**) *G. miyajii* Tos.Takah. sp. nov. strain Thu10. (**f** ) *G. bhattacharyae* Tos.Takah. sp. nov. strain 118.

Using LV FE-SEM, we detected cellulose filaments of the mother cell wall on the surface of *Glaucocystis* colonies (Fig. 2). The fibrils on the colony surface were essentially identical in shape among the strains examined, but two types of filament arrangements were recognised. The entire colony surface generally exhibited a gauze fabric-like appearance, with small spaces between fibrils, in *G. geitleri* E.G.Pringsh. *ex* Tos.Takah. & Nozaki sp. nov., *G. nostochinearum*, *G. oocystiformis* and *G. miyajii* Tos.Takah. & Nozaki sp. nov. (Fig. 2a,b,d,e). On the other hand, the fibrils were tightly arranged, with no spaces between them, over nearly the entire colony surface in *G. incrassata* (Lemmerm.) Tos.Takah. & Nozaki stat. nov. and *G. bhattacharyae* Tos.Takah. & Nozaki sp. nov. (Fig. 2c,f).

This ultrastructural difference in the mother cell wall (Fig. 2) is consistent with the difference in expansion of the mother cell wall observed under LM (Fig. 1; Table 1; Supplementary Note).

### Ultra-high-voltage electron microscopy and ultrathin-section transmission electron microscopy

Recent reports^13^,^14^ using UHVEM tomography clearly revealed the 3D ultrastructural features of the plasma membrane and the flattened vesicles at the protoplast periphery in three strains or species of *Glaucocystis* (*G. geitleri* strain SAG 229-1, *G. nostochinearum* strain SAG 16.98 and *G. incrassata* strain SAG 229-2), even though the protoplast was tightly enclosed by a cell wall. In addition, the 3D ultrastructures of the protoplast periphery in these three strains are diverse and can be classified into three types (periphery types A, B and C)^13^,^14^. Since these three strains were found to represent three different species (*G. geitleri* strain SAG 229-1 of type A, *G. nostochinearum* strain SAG 16.98 of type B and *G. incrassata* strain SAG 229-2 of type C), they were assigned as authentic strains for these species (see below).

**Figure 3:**
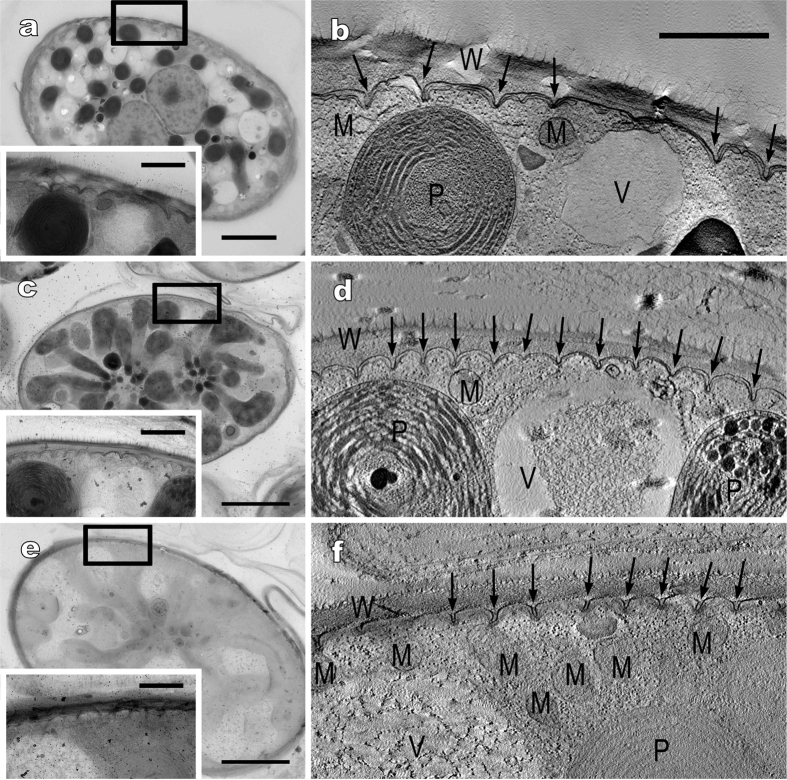
Electron tomography of protoplast periphery of vegetative cells of *G. oocystiformis* strain 126 (**a,b**), *G. miyajii* Tos.Takah. & Nozaki sp. nov. strain Thu10 (**c,d**) and *G. bhattacharyae* Tos.Takah. & Nozaki sp. nov. strain 118 (**e,f**). Note that *G. oocystiformis* exhibits periphery type A whereas *G. miyajii* and *G. bhattacharyae* exhibit periphery type C (Supplementary Fig. 4). See also Supplementary Videos 1–3. (**a,c,e**) Ultra-high voltage electron microscopic images. Insets show higher magnification image of the cell periphery (boxed area). Scale bar, 5 μm and 1 μm (insets). (**b,d,f** ) Tomographic images of boxed area in (**a,c,e**), respectively. Shown at the same magnification. Scale bar, 1 μm. M, mitochondrion; P, plastid; V, vacuole; W, cell wall. Arrows indicate bar-like grooves of plasma membrane covered by invaginations of flattened vesicles.

**Figure 4:**
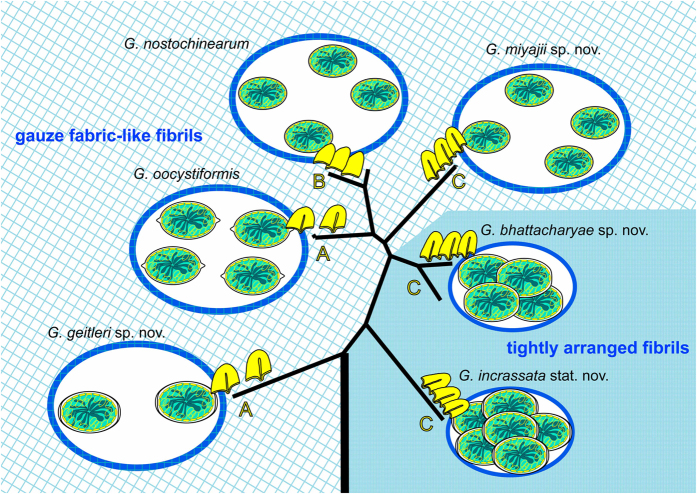
Diagram of the novel *Glaucocystis* taxonomic system based on the present study. Six *Glaucocystis* (*G.*) species were classified based on morphological characteristics and molecular phylogeny of cultured material. Colony surface (blue) exhibit two cellulose fibril types under FE-SEM (Fig. 2): gauze fabric-like fibrils (left) and tightly arranged fibrils (right), indicated by two background designs. Periphery types A–C are distinguishable under UHVEM and TEM (Supplementary Fig. 4)^13^,^14^, indicated by enlarged diagram of flattened vesicles (yellow) in each species. Four types of cell wall shape at the cell poles are recognised under LM (Supplementary Fig. 1). Phylogeny is based on phylogenetic tree of the concatenated gene sequences (Supplementary Fig. 5); each species exhibits sufficient genetic distances from other species (Supplementary Figs 6–8).

To examine the peripheral 3D ultrastructure of protoplasts in the other three *Glaucocystis* species, we observed various regions of mature vegetative cells in three strains representing the three species (*G. oocystiformis* strain 126, *G. miyajii* strain Thu10 and *G. bhattacharyae* strain 118, designated here as the authentic strains for the three species; see below) by UHVEM and tomography, as well as ultrathin section TEM (Table 1; Fig. 3; Supplementary Videos 1–3; Supplementary Fig. 3). The protoplast periphery of these species was similar to that in the former three species examined previously. The flattened vesicles were leaflet-like in shape, lacked a plate-like interior structure, and were distributed throughout the entire protoplast periphery just underneath the single-layered plasma membrane (except for the region near basal bodies), but they did not completely enclose the protoplast periphery to form small spaces between the vesicles at the protoplast periphery. In addition, based upon the present UHVEM tomography, *G. oocystiformis* strain 126 was assigned to periphery type A, whereas *G. miyajii* strain Thu10 and *G. bhattacharyae* strain 118 were assigned to periphery type C. Based on the native 3D ultrastructural features of the protoplast periphery established by previous and present studies using UHVEM tomography^13^,^14^, the three periphery types are evident and distinguishable from each other, even based on ultrathin-section TEM alone. Thus, peripheral protoplast types were determined in other strains based on ultrathin-section TEM alone; each of the six species exhibited only a single periphery type, despite being composed of more than one strain. *G. nostochinearum* (periphery type B) and *G. miyajii* (periphery type C) were clearly distinguished from each other based on the difference in the periphery type, although they were indistinguishable under LM alone (Table 1; Figs 1 and 4; Supplementary Fig. 1).

### Molecular phylogenetic analyses

The phylogenetic tree of the concatenated plastid gene sequences (Supplementary Fig. 5) demonstrated that 13 *Glaucocystis* strains could be subdivided into six phylogenetic groups [four robust monophyletic groups and two independent operational taxonomic units (OTUs)], which are essentially equivalent to the G1–G6 groups recognised previously^31^. These six groups corresponded to the six species delineated by our comparative morphological analysis (Table 1).

In the phylogenetic tree, basal phylogenetic relationships were robustly resolved (with bootstrap values of 82–100%; Supplementary Fig. 5); *G. geitleri* and *G. incrassata* occupied the most and second most basal positions, respectively, whereas the other four species (*G. nostochinearum*, *G. oocystiformis*, *G. miyajii* and *G. bhattacharyae*) represent a large robust monophyletic group (crown lineage), supported by bootstrap values of 100% in neighbour-joining (NJ) and maximum likelihood (ML) analyses. However, the phylogenetic relationships of the four species were not well resolved within the crown lineage.

### Internal transcribed spacer-2 secondary structure and genetic distances

The six species of *Glaucocystis* were evaluated by compensatory base changes (CBCs) in the secondary structure of the internal transcribed spacer (ITS)-2 of nuclear ribosomal DNA (*r*DNA) (Supplementary Figs 6 and 7) and the genetic distances of a plastid gene (Supplementary Fig. 8). Four *Glaucocystis* species within the crown lineage exhibited CBCs and sufficient genetic distances to be classified as four distinct species (Supplementary Note).

## Discussion

Based on the present comparative morphological and molecular examinations of cultured materials from the genus *Glaucocystis*, six species were clearly delineated (Table 1; Fig. 4). In contrast to previous reports^8^,^9^, that 45 to 50 years ago had to rely on conventional EM only, ultrastructural diversity of the protoplast periphery was significant within the genus *Glaucocystis* when examined by UHVEM (Figs 3 and 4; Supplementary Figs 3 and 4). Moreover, ultrastructural diversity was clarified in the arrangement of cellulose filaments of the mother cell under LV FE-SEM (Figs 1 and 2). Based on the differences in these new-generation EM characteristics and LM features of the 13 *Glaucocystis* strains, we could delineate six morphological species that correspond to six phylogenetic groups (G1–G6) recognised by previous^31^ and present phylogenetic analyses (Table 1; Fig. 4; Supplementary Fig. 5). Although *G. oocystiformis* can be easily distinguished from the other five species based on differences in LM characteristics and phylogenetic positions (Table 1; Fig. 1; Supplementary Figs 1 and 5), the *in situ* ultrastructural features of *G. oocystiformis* are similar to those of *G. geitleri* in having periphery type A and gauze fabric-like fibrils (Supplementary Fig. 5). Among the other four species, *G. bhattacharyae* and *G. incrassata* have essentially the same *in situ* ultrastructures (periphery type C and tightly arranged fibrils), although they were distinguished from one another based on differences in LM characteristics (Table 1; Supplementary Fig. 1). Thus, in addition to new-generation EM observations, LM data and molecular phylogenetic analyses are essential for delineating microalgal species.

The novel strains established here from a single field sample were classified into three species (Table 1; Supplementary Table 1; Supplementary Fig. 5). Although *G. nostochinearum* has been considered a cosmopolitan species^24^,^32^,^33^, the records may be based on several species, which can be distinguished using the new-generation taxonomic methodology established here (Supplementary Note).

The plasma membrane of five of the six *Glaucocystis* species had numerous grooves throughout the protoplast surface (Figs 3 and 4; Supplementary Figs 3 and 4)^13^,^14^. In *G. nostochinearum*^13^, however, the plasma membrane lacked grooves or invaginations (type B; Supplementary Fig. 4) as in *Cyanophora* species^10^,^11^. Since *G. nostochinearum* belongs to the crown lineage within *Glaucocystis* (Supplementary Fig. 5), the lack of grooves or invaginations in *G. nostochinearum* might have evolved secondarily within *Glaucocystis* (Fig. 4). Vegetative cells of *Cyanophora* species are apparently smaller than those of *Glaucocystis*^10^, and *G. nostochinearum* exhibits one of the smallest cell sizes within *Glaucocystis* (Table 1; Fig. 1; Supplementary Fig. 1). In addition, the surface-area-to-volume ratio is smaller (inversely proportional to the cell size) and the transportation of substances across the plasma membrane more limited in larger cells. Thus, the presence of grooves or invaginations at the protoplast periphery in the five *Glaucocystis* species might contribute to expansion of the surface area of the protoplast and consequently to their large cell size.

The Gloeochaetales are another order of glaucophytes that are characterised by having palmelloid immotile vegetative cells and include two genera, *Gloeochaete* and *Cyanoptyche*^20^; some species have zoospores^34^,^35^,^36^. These algae might represent the evolutionarily intermediate stage between the flagellate *Cyanophora* (Cyanophorales) and the coccoid *Glaucocystis* (Glaucocystales). Within the Gloeochaetales, cultured strains labelled *Cyanoptyche gloeocystis* Pascher^37^ and *Gloeochaete wittrockiana* Lagerh^38^ are available, but do not number more than three in each species ( http://www.ccac.uni-koeln.de/; http://sagdb.uni-goettingen.de/). Although no taxonomic studies have been performed based on EM and/or molecular data, these two taxa are considered cosmopolitan species^34^,^39^. Therefore, taxonomic studies based on molecular methods and comparative *in situ* ultrastructural characteristics using various clonal strains, as in *Cyanophora*^10^ and *Glaucocystis* evaluated here, would be useful for these two species or genera. 3D UHVEM tomography will reveal the peripheral *in situ* ultrastructures of their vegetative cells even when enclosed by a non-cellulosic extracellular matrix^35^,^36^, as in *Glaucocystis*^13^,^14^. LV FE-SEM may be applicable in easily inducible, naked zoospores of the Gloeochaetales^35^,^36^ for *in situ* ultrastructural observation of the protoplast surface, as in *Cyanophora*^10^. These two types of new-generation EM should be capable of revealing the actual diversity in ultrastructures in the peripheral protoplast *in situ*, leading to the delineation of more natural species of the gloeochaetalean algae, when combined with molecular phylogenetic results.

## Conclusions

In recent taxonomic work on certain microorganisms, there has been a tendency to avoid morphological approaches in favour of molecular ones^15^,^16^,^17^,^18^. However, species delineation based only on molecular data cannot demonstrate how the species live. Even when whole-genome sequence data are available, we can only speculate on the metabolic pathways employed by the organism. Even in bacteria/archaea, species delineation has been carried out on the basis of phenotypic characteristics^40^,^41^. Next-generation microbial taxonomy, which is just now becoming established, utilises new-generation EM methods (e.g. FE-SEM and UHVEM) to demonstrate detailed *in situ* ultrastructural features of microscopic organisms in their entirety. Molecular barcoding is only meaningful for lineages within which species have already been delineated and recognised by morphological or phenotypic characteristics. Global and *in situ* ultrafine microscopy should become the mainstream method used to delineate microbial species, as in the present study on *Glaucocystis*.

## Taxonomic Accounts

***Glaucocystis nostochinearum*** Itzigs. *ex* Rabenh. (in Alg. Eur. 94–5: no. 1935. 1866)^21^,^22^.

*Syntypes*: Rabenhorst’s exsiccata, *Die Algen Europas*^21^ packet no. 1935.

*Lectotype (here designated)*: the permanent slide R1935J prepared from a syntype of Farlow Herbarium, University of Harvard (FH), deposited in FH (Supplementary Fig. 2; Supplementary Note).

*Syntypic authentic strain*: not available.

*Type locality*: Berlin, Prussia (now, Germany).

*Epitype (here designated)*: Resin-embedded cells of the new authentic strain SAG 16.98, deposited as TNS-AL-58925 in Department of Botany, National Museum of Nature and Science (TNS).

*Epitypic authentic strain (here designated)*: SAG 16.98.

*Epitype locality*: Lower Saxony, pond in quarry at Walkenried/Harz, surface of *Myriophyllum* sp., Germany.

***Glaucocystis oocystiformis*** Prescott (in Farlowia 1(3): 372. 1944)^23^.

*Holotype*: Prescott 1944. Farlowia. pl. 4, Fig. 20.

*Holotypic authentic strain*: not available.

*Type locality*: Trout Lake, Vilas County, Wisconsin, USA.

*Epitype (here designated)*: Resin-embedded cells of the new authentic strain 126, deposited as TNS-AL-58926 in TNS.

*Epitypic authentic strain (here designated)*: Isolate 126, also available as NIES-3868 (Supplementary Table 1).

*Epitype locality*: Funabashi-shi, Chiba, Japan (35.694283°N, 140.048166°E).

***Glaucocystis incrassata*** (Lemmerm.) Tos.Takah. & Nozaki **stat. nov.**

*Basionym: **Glaucocystis nostochinearum*** **var.** ***incrassata*** Lemmerm. (in Arch. Hydrobiol. Planktonkd. 4: 178. 1908)^42^.

*Holotype*: Lemmermann, Arch. Hydrobiol. Planktonkd. 4: 178. 1908, Taf. V., Fig. 4^42^.

*Holotypic authentic strain*: not available.

*Type locality*: Lentini, Sicily, Italy.

*Epitype (here designated)*: Resin-embedded cells of the new authentic strain SAG 229-2, deposited as TNS-AL-58923 in TNS.

*Epitypic authentic strain (*here designated*)*: SAG 229-2.

*Epitype locality*: Denmark.

***Glaucocystis geitleri*** E.G.Pringsh. *ex* Tos.Takah. & Nozaki **sp. nov.**

≡***Glaucocystis geitleri*** E.G.Pringsh. (in Stud. Pl. Physiol. 1958)^43^ *nom. provis., inval.* (see Supplementary Note).

*Diagnosis*:

Coccoid alga, enclosed by cellulosic cell wall; solitary or colonial generally with two cells. Cells *ca*. 30–40 μm long × 20–30 μm wide, truncate-ellipsoidal, often with clear polar thickenings, lacking polar nodules and equatorial ring. Two vestigial flagella between cell wall and protoplast periphery, positioned at equator of cells. Protoplast periphery, with numerous small depressions arranged regularly. Depression at intervals of *ca*. 500–800 nm, shared by plasma membrane and centre of underlying flattened vesicle. Flattened vesicles leaflet-like, not overlapping one another. Colony lacking attaching stalk; mother cell wall extended prominently, with a gauze fabric-like appearance and small spaces between fibrils.

*Type locality*: Cambridge, England, UK.

*Holotype*: Resin-embedded cells of the new authentic strain SAG 229-1, deposited as TNS-AL-58922 in TNS.

*Holotypic authentic strain*: SAG 229-1.

***Glaucocystis miyajii*** Tos.Takah. & Nozaki **sp. nov.**

*Diagnosis*:

Coccoid alga, enclosed by cellulosic cell wall; solitary or colonial generally with four cells. Cells *ca*. 19–24 μm long × 10–15 μm wide, ellipsoidal, lacking polar thickenings, polar nodules and equatorial ring. Two vestigial flagella between cell wall and protoplast periphery, positioned at equator of cells. Protoplast periphery, with numerous small depressions arranged regularly. Depression at intervals of *ca*. 200–600 nm, shared by plasma membrane and centre of underlying flattened vesicle. Flattened vesicles leaflet-like, slightly overlapping one another. Colony lacking attaching stalk; mother cell wall extended prominently, with a gauze fabric-like appearance and small spaces between fibrils.

*Type locality*: Funabashi-shi, Chiba, Japan (35.694283°N, 140.048166°E).

*Holotype*: Resin-embedded cells of the new authentic strain Thu10, deposited as TNS-AL-58924 in TNS.

*Holotypic authentic strain*: Isolate Thu10, also available as NIES-3867 (Supplementary Table 1).

*Etymology*: Named after Prof. Kazuyuki Miyaji (University of Toho), who contributed much to phycology.

***Glaucocystis bhattacharyae*** Tos.Takah. & Nozaki **sp. nov.**

*Diagnosis*:

Coccoid alga, enclosed by cellulosic cell wall; solitary or colonial generally with four cells. Cells *ca*. 17–27 μm long × 12–22 μm wide, truncate-ellipsoidal, lacking polar thickenings, polar nodules and equatorial ring. Two vestigial flagella between cell wall and protoplast periphery, positioned at equator of cells. Protoplast periphery, with numerous small depressions arranged regularly. Depression at intervals of *ca*. 200–600 nm, shared by plasma membrane and centre of underlying flattened vesicle. Flattened vesicles leaflet-like, slightly overlapping one another. Colony lacking attaching stalk; mother cell wall lacking prominent extension and a gauze fabric-like appearance, with tightly arranged fibrils and no spaces between fibrils.

*Type locality*: Funabashi-shi, Chiba, Japan (35.694283°N, 140.048166°E).

*Holotype*: Resin-embedded cells of the new authentic strain 118, deposited as TNS-AL-58921 in TNS.

*Holotypic authentic strain*: Isolate 118, also available as NIES-3866 (Supplementary Table 1).

*Etymology*: Named after Prof. Debashish Bhattacharya (Rutgers University), who contributed much to phycology.

### Key to species of *Glaucocystis*

Based on Table 1 and Supplementary Table 2.

A. Colony with stalk-----------------------------------------------------------------------------B.

A. Colony without stalk--------------------------------------------------------------------------C.

B. Cell shape ellipsoidal-----------------------------------------------------*G. indica* R.J.Patel

B. Cell shape kidney-shaped--------------------------------------------------------------------------------------------------------------*G. reniformis* B.N.Prasad, R.K.Mehrotra & P.K.Misra

C. Cell wall with equatorial ring-------------------------------------------*G. cingulata* Bohlin

C. Cell wall without equatorial ring------------------------------------------------------------D.

D. Cell spherical--------------------------------------------------------------*G. duplex* Prescott

D. Cell ellipsoidal---------------------------------------------------------------------------------E.

E. Cell measured 10–18 × 6–10 μm-------------------------------------------*G. bullosa* Wille

E. Cell measured 17–50 × 10–30 μm----------------------------------------------------------F.

F. Cell wall with polar nodules--------------------------------------*G. oocystiformis* Prescott

F. Cell wall without polar nodules--------------------------------------------------------------G.

G. Cell wall with polar thickenings------------------------------------------------------------H.

G. Cell wall without polar thickenings----------------------------------------------------------I.

H. Mother cell wall with prominent extension, having a gauze fabric-like appearance and small spaces between fibrils; cell measured 30–50 × 19–30 μm; grooves at intervals of 500–800 nm; vesicles not overlapping---------------------------------------------------------------------------------------------*G. geitleri* E.G.Pringsh. *ex* Tos.Takah. & Nozaki sp. nov.

H. Mother cell wall without prominent extension, lacking a gauze fabric-like appearance and spaces between fibrils; cell measured 22–32 × 15–24 μm; grooves at intervals of 200–600 nm; vesicles frequently overlapping-----------------------------------------------------------------------*G. incrassata* (Lemmerm.) Tos.Takah. & Nozaki stat. nov.

I. Poles of cell truncate; mother cell wall without prominent extension, lacking a gauze fabric-like appearance and spaces between fibrils---------------------------------------------------------------------------------------------*G. bhattacharyae* Tos.Takah. & Nozaki sp. nov.

I. Poles of cell not truncate; mother cell wall with prominent extension, having a gauze fabric-like appearance and small spaces between fibrils-------------------------------------J.

J. Protoplast periphery with grooves-------------*G. miyajii* Tos.Takah. & Nozaki sp. nov.

J. Protoplast periphery without grooves------------*G. nostochinearum* Itzigs. *ex* Rabenh.

## Methods

### Strains and culture conditions for observation

Ten culture strains of *Glaucocystis* were obtained from public culture collections (Supplementary Table 1) at the National Institute for Environmental Studies (NIES, http://mcc.nies.go.jp/)^44^ and the Sammlung von Algenkulturen der Universität Göttingen (SAG, http://sagdb.uni-goettingen.de/)^45^,^46^. We also used three strains of *Glaucocystis* newly established from freshwater samples collected in Japan (strains 118, 126 and Thu10; Supplementary Table 1). The cultures were maintained as described previously^13^.

### Light microscopy

Permanent slides were prepared using air-dried cells from the syntype material of *G. nostochinearum* and cultured material from authentic strains. Rehydrated cells from the syntype material or cultured cells were fixed with 2% glutaraldehyde in medium and washed with medium and distilled water on 0.1%-poly-L-lysine-coated 18-mm micro-cover glasses (Matsunami Glass Ind., Ltd., Kishiwada, Japan). After dehydration using a graded ethanol series and infiltration with xylene, the glass was covered with 60 °C Canada balsam xylene, placed on the 60 °C Canada balsam on a glass slide, and then the slide was incubated at 60 °C for a few days. LM observations were carried out as described previously^10^ using the permanent slides and living cultured cells.

### Field-emission scanning electron microscopy

LV FE-SEM was performed as described previously^11^ using all 13 *Glaucocystis* strains, but cells were harvested directly, treated with the critical point dryer JCPD-5 (JEOL) and observed using the UHR FE-SEM SU8220 (Hitachi High-Technologies, Tokyo, Japan).

### Transmission electron microscopy and ultra-high-voltage electron microscopy

Since the high-pressure freezing and freeze-substitution fixation method is generally expected to be superior to chemical fixation in preserving the integrity of cellular ultrastructures^12^, this method was performed for TEM and UHVEM as described previously^13^. Ultrathin-section TEM was also performed as described previously^13^ for all 13 *Glaucocystis* strains. In addition, UHVEM and reconstruction of the tomographic images were carried out as described previously^13^ in three authentic strains of three *Glaucocystis* species (Fig. 3).

### Molecular phylogenetic analysis and comparative analysis of the secondary structure of ITS-2 in nuclear ribosomal DNA

DNA extraction, polymerase chain reaction (PCR) and direct sequencing of the PCR products were performed as described previously^10^,^47^,^48^, using primers designed in a previous study^10^,^31^,^49^. The secondary structure of nuclear *r*DNA ITS-2 was constructed as described previously^10^. Phylogenetic relationships between *Glaucocystis* species were examined based on analyses of the concatenated sequences (2,211 base pairs) of the photosystem I P700 chlorophyll *a* apoprotein A2 (*psaB*) gene (1,461 base pairs) and the photosystem II P680 chlorophyll *a* apoprotein D1 (*psbA*) gene (750 base pairs) from 13 strains of *Glaucocystis*, representing 10 OTUs (based on identical sequences), and three strains of three other glaucophyte genera as an outgroup (Supplementary Table 1). The sequences were aligned as described previously^10^ and subjected to phylogenetic analyses. ML and NJ analyses were performed as described previously^10^, except that one selected model was used: the general time reversible + gamma model with invariant sites for ML.

## Additional Information

**How to cite this article**: Takahashi, T. *et al.* Delineation of six species of the primitive algal genus *Glaucocystis* based on *in situ* ultrastructural characteristics. *Sci. Rep.* **6**, 29209; doi: 10.1038/srep29209 (2016).

## Supplementary Material

Supplementary Information

Supplementary Video 1

Supplementary Video 2

Supplementary Video 3

## Figures and Tables

**Table 1:** Comparison of the morphological characteristics of six *Glaucocystis* species delineated in the present study.

Species	*G. oocystiformis* Prescott	*G. geitleri* sp. nov.^a^	*G. incrassata* stat. nov.^a^	*G. bhattacharyae* sp. nov.^a^	*G. miyajii* sp. nov.^a^	*G. nostochinearum* Itzigs. *ex* Rabenh.^a^
Mother cell wall extension	prominent	prominent	not prominent	not prominent	not prominent	not prominent
Gauze fabric-like appearance of mother cell wall	present	present	absent	absent	present	present
Cell numbers within a colony	2–4, generally 4	2–4, generally 2	4–8, generally 4	1–4, generally 4	2–4, generally 4	2–4, generally 4
Cell size	*ca*. 15–25 μm wide × *ca*. 25–35 μm long	*ca*. 20–30 μm wide × *ca*. 30–40 μm long	*ca*. 13–23 μm wide × *ca*. 20–30 μm long	*ca*. 12–22 μm wide × *ca*. 17–27 μm long	*ca*. 10–15 μm wide × *ca*. 19–24 μm long	*ca*. 10–17 μm wide × *ca*. 18–27 μm long
Cell and polar shape	ellipsoidal with polar nodule	truncate-ellipsoidal with polar thickening	truncate-ellipsoidal with polar thickening	truncate-ellipsoidal without polar thickening	ellipsoidal without polar nodule	ellipsoidal without polar nodule
Cell wall thickness	*ca*. 150–350 nm	*ca*. 300–500 nm	*ca*. 100–300 nm	*ca*. 150–350 nm	*ca*. 150–350 nm	*ca*. 100–300 nm
Protoplast periphery^b^	type A	type A	type C	type C	type C	type B
Regular groove	present	present	present	present	present	absent
Groove interval	*ca*. 500–800 nm	*ca*. 500–800 nm	*ca*. 200–600 nm	*ca*. 200–600 nm	*ca*. 200–600 nm	ND
Vesicle frequent overlapping	present	present	absent	absent	absent	present
Authentic strain	126	SAG 229-1	SAG 229-2	118	Thu10	SAG 16.98
Other strains examined	NIES-1369, NIES-966	SAG 229-3, SAG 28.80		SAG 27.80	NIES-1961	SAG 45.88
Corresponding phylogenetic group resolved in previous study^31^	G4	G1	G6	G5	G3	G2

^a^Species that could be identified as *G. nostochinearum* based on the traditional taxonomic concept (Supplementary Table 2).

^b^See Supplementary Fig. 4.
