# Supplementary material for: Delineation of six species of the primitive algal genus Glaucocystis based on in situ ultrastructural characteristics
Source: Sci Rep. 2016 Jul 7;6:29209. doi: 10.1038/srep29209 (PMC4935853; doi:10.1038/srep29209)
Supplement: Supplementary Information [file srep29209-s1.pdf]

Supplementary Information

Delineation of six species of the primitive algal genus *Glaucocystis* based on *in situ* ultrastructural characteristics

Toshiyuki Takahashi, Tomoki Nishida, Akihiro Tuji, Chieko Saito, Ryo Matsuzaki, Mayuko Sato, Kiminori Toyooka, Hidehiro Yasuda & Hisayoshi Nozaki*

Affiliations

Department of Biological Sciences, Graduate School of Science, University of Tokyo, 7-3-1 Hongo, Bunkyo-ku, Tokyo, 113-0033, Japan

Toshiyuki Takahashi, Chieko Saito, Ryo Matsuzaki & Hisayoshi Nozaki*

Research Center for Ultra-High Voltage Electron Microscopy, Osaka University, 7-1 Mihogaoka, Ibaraki, Osaka, 567-0047, Japan

Tomoki Nishida & Hidehiro Yasuda

Department of Botany, National Science Museum, 4-1-1 Amakubo, Tsukuba, Ibaraki, 305-0005, Japan

Akihiro Tuji

RIKEN Center for Sustainable Resource Science, 1-7-22 Suehiro-cho, Tsurumi-ku, Yokohama, Kanagawa, 230-0045, Japan

Mayuko Sato & Kiminori Toyooka

Contact information

Correspondence to: Hisayoshi Nozaki*

e-mail: nozaki@bs.s.u-tokyo.ac.jp

Supplementary Table 1. Species and strains of *Glaucocystis* and other glaucophytes used in this study.

Species	Strain designation	Origin of strain	Locality	GenBank accession number		
				psaB	*psbA*	ITS1-5.8S *r*DNA-ITS2
Glaucocystis nostochinearum	SAG[a] 16.98 (new epitypic authentic strain)	Pond in quarry at surface of *Myriophyllum* spec.[a]	Walkenried/Harz, Lower Saxony, Germany (51°35'33.5"N 10°36'10.0"E)[a]	AB973457	KF631337	*LC120721*
	SAG 45.88	Pool at Voslapp, desalted sand from Jade River[a]	Voslapp, Wilhelmshaven, Germany (53°35'24.6"N 8°06'29.0"E)[a]	*LC120666*	KF631335	*LC120722*
Glaucocystis miyajii	Thu10[b,c] (holotypic authentic strain) (=NIES[d]-3867)	Freshwater sample from a pond[c]	Miyama, Funabashi-shi, Chiba, Japan, in 3 July 2012 (35°41'43.3"N 140°02'53.8"E)	*LC120667*	*LC120683*	*LC120723*
	NIES-1961	Freshwater[d]	Kofutamata-machi, Kanazawa-shi, Ishikawa, Japan[d]	*LC120668*	KF631333	*LC120724*
Glaucocystis bhattacharyae	118[b,c] (holotypic authentic strain) (=NIES-3866)	Freshwater sample from a pond[c]	Miyama, Funabashi-shi, Chiba, Japan, in 3 July 2012 (35°41'43.3"N 140°02'53.8"E)	*LC120669*	*LC120684*	*LC120725*
	SAG 27.80	Freshwater[a]	France[a]	*LC120670*	KF631328	*LC120726*
Glaucocystis incrassata	SAG 229-2 (new epitypic authentic strain)	Freshwater[a]	Denmark[a]	*LC120671*	KF631325	*LC120727*
Glaucocystis geitleri	SAG 229-1 (holotypic authentic strain) (=NIES-2141)	Freshwater[a]	Cambridge, England, UK (52°11'32.5"N 0°09'48.2"E)[a,d]	AB973458	*LC120685*	*LC120728*
	SAG 229-3	Freshwater[a]	ND[a]	*LC120673*	KF631327	*LC120729*
	SAG 28.80	Freshwater[a]	ND[a]	*LC120674*	KF631326	*LC120730*
Glaucocystis oocystiformis	126[b,c] (new epitypic authentic strain) (=NIES-3868)	Freshwater sample from a pond[c]	Miyama, Funabashi-shi, Chiba, Japan, in 3 July 2012 (35°41'43.3"N 140°02'53.8"E)	*LC120675*	*LC120686*	*LC120718*
	NIES-1369	Freshwater[d]	Kakuma-machi, Kanazawa-shi, Ishikawa, Japan[d]	*LC120676*	KF631330	*LC120719*
	NIES-966	Freshwater from Renge-numa[d]	Kitashiobara-mura, Yama-gun, Fukushima, Japan[d]	*LC120677*	KF631329	*LC120720*
Cyanophora paradoxa[f]	CCAP[e] 981/1[f] (epitypic authentic strain)			AB973446	*LC120678*	

Cyanophora sudae	NIES-764[f] (holotypic authentic strain)	AB973453	KF631321
"*Gloeochaete wittrockiana*"	SAG 46.84	AB973454	KF631340
"*Cyanoptyche gloeocystis*"	SAG 34.90	AB973456	KF631339

Accession numbers in italics type indicate sequences determined by this work.

[a] Sammlung von Algenkulturen der Universität Göttingen (SAG, http://sagdb.uni-goettingen.de/)[1].

[b] Newly isolated in this study.

[c] From this sample, 16 clonal isolates were established using the pipette-washing method. Base on the *psaB* and nuclear ITS1-5.8S *r*DNA-ITS2 sequences, these new cultures were divided into three genetic groups: 118 group (118, 115, 116, 119, 123, 131); 126 group (126, 134, 121, 124, 125, 128, 127, 130, Thu9) and Thu10 group (Thu10). Therefore, from each group, only a single isolate was chosen and used for this study.

[d] National Institute for Environmental Studies (NIES, http://mcc.nies.go.jp/)[2].

[e] Culture Collection of Algae and Protozoa (CCAP, http://www.ccap.ac.uk/).

[f] For details of the strains and species information, see reference[3].

Supplementary Table 2. Comparison of the morphological characteristics of *Glaucocystis* species based on the traditional taxonomic concept.

Species	*G. bullosa* Wille	*G. indica* R.J.Patel	*G. reniformis* B.N.Prasad *et al.*	*G. cingulata* Bohlin	*G. duplex* Prescott	*G. nostochinearum* Itzigs. *ex* Rabenh.	*G. oocystiformis* Prescott
Mother cell wall expansion	ND	prominent	prominent	prominent	prominent	prominent	prominent
Formation of colony stalks	absent	present	present	absent	absent	absent	absent
Cell numbers within a colony	3–8	2–8	2–4, generally 2	2–8	8–16	2–8	2–16
Cell size	*ca*. 6–10 µm wide × *ca*. 10–18 µm long	*ca*. 9–18 µm wide × *ca*. 18–31 µm long	*ca*. 15–18 µm wide × *ca*. 24–29 µm long	*ca*. 12–68 µm wide × *ca*. 16–68 µm long	*ca*. 40–44 µm wide × *ca*. 40–44 µm long	*ca*. 10–24 µm wide × *ca*. 18–32 µm long	*ca*. 17–36 µm wide × *ca*. 27–53 µm long
Cell and polar shape	ellipsoidal	ellipsoidal	kidney-shaped	ellipsoidal to spherical	spherical	ellipsoidal	ellipsoidal with polar nodule
Equatorial ring	absent	absent	absent	present	absent	absent	absent
Cell wall thickness	absent	absent	absent	absent	absent	absent or present	present
Authentic strains	not available	not available	not available	not available	not available	here designated	here designated
Based on	original description[4,5]	original description[6]	original description[7]	original description[8], reference[9,10]	original description[11], reference[9,10,12]	original description[13,14], reference[9,10,12]	original description[11], reference[9,10,12]

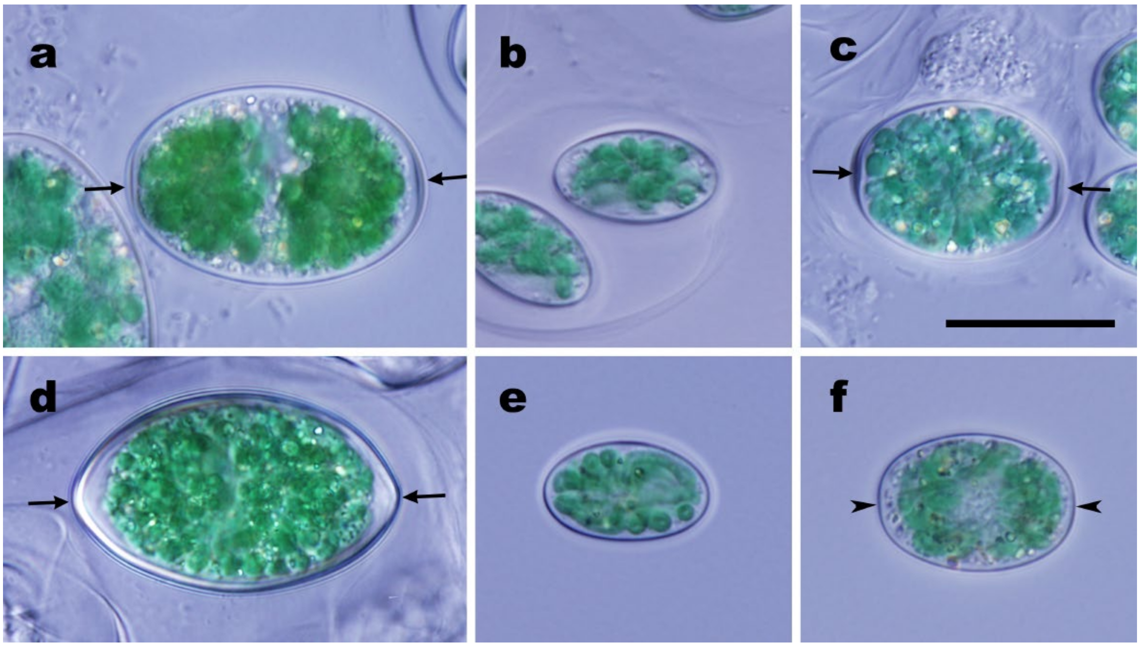

Supplementary Figure 1 | Differential interference contrast microscopy of vegetative cells of six *Glaucocystis* species. Shown at the same magnification. Scale bar, 20 µm. Note that each immobile vegetative cell is enclosed tightly by a cell wall. (a) *G. geitleri* E.G.Pringsh. *ex* Tos.Takah. & Nozaki sp. nov. strain SAG 229-1, showing thickenings of cell wall at truncated cell poles (arrows). (b) *G. nostochinearum* Itzigs. *ex* Rabenh. strain SAG 16.98. (c) *G. incrassata* (Lemmerm.) Tos.Takah. & Nozaki stat. nov. strain SAG 229-2, showing thickenings of cell wall at truncated cell poles (arrows). (d) *G. oocystiformis* Prescott strain 126, showing polar nodules (arrows). (e) *G. miyajii* Tos.Takah. & Nozaki sp. nov. strain Thu10. (f) *G. bhattacharyae* Tos.Takah. & Nozaki sp. nov. strain 118, showing truncate cell poles without thickening of cell wall (arrowheads).

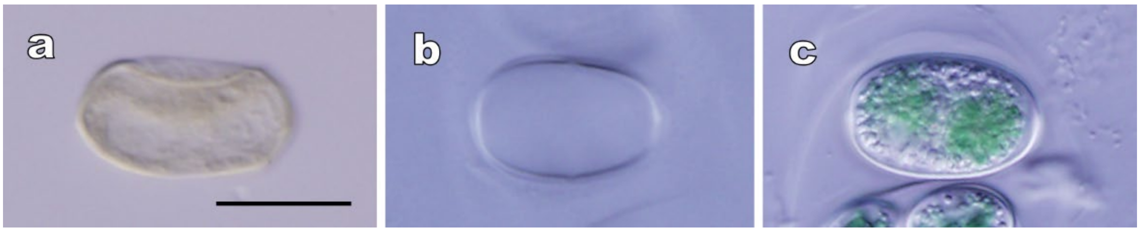

Supplementary Figure 2 | Differential interference contrast microscopy of vegetative cells of *Glaucocystis nostochinearum* Itzigs. *ex* Rabenh. Shown at the same magnification. Scale bar, 20 µm. (a) A cell in the permanent slide R1935J (lectotype designated in the present study) prepared using syntype material[13,14] from FH, showing ellipsoidal cell shape without distinctive characters in the cell wall at cell poles. (b) A cell in the permanent slide prepared using epitypic authentic strain SAG 16.98 of *G. nostochinearum*. (c) A living cell of the authentic strain SAG 16.98 of *G. nostochinearum*.

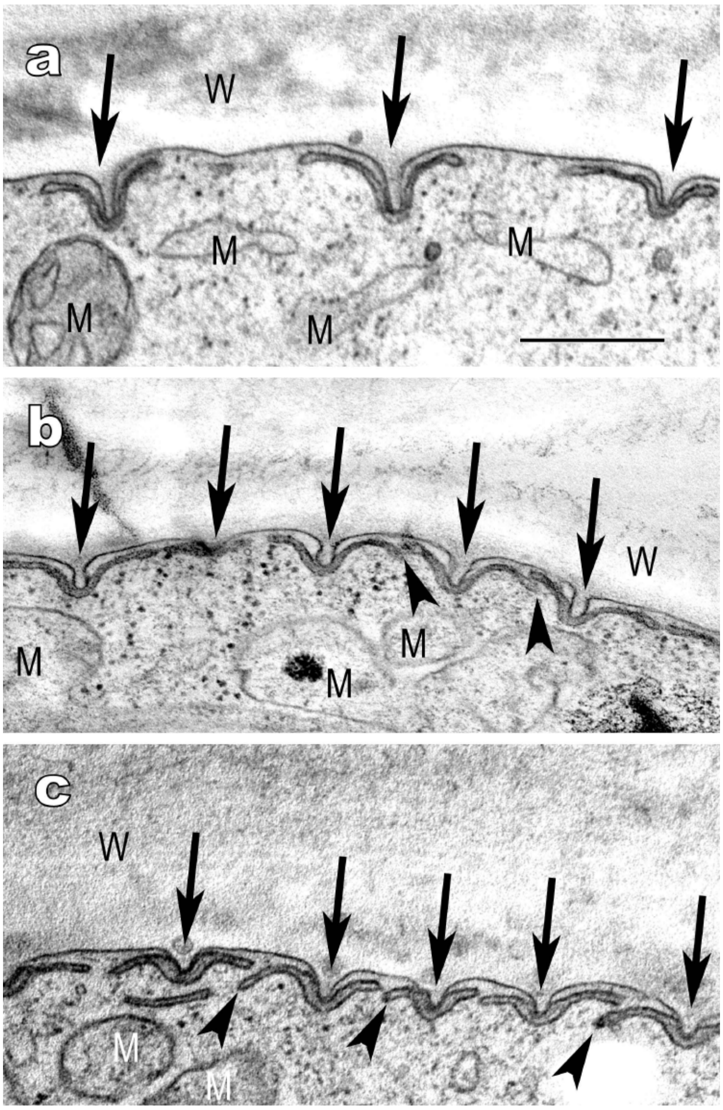

Supplementary Figure 3 | Ultrathin section transmission electron microscopy of three *Glaucocystis* species. Shown at the same magnification. Scale bar, 500 nm. M, mitochondrion; S, starch; V, vacuole; W, cell wall. Note that the cell periphery consists of plasma membrane and underlying flattened vesicles lacking plate-like structure inside both of which are grooved (arrows). The vesicles are slightly overlapping one another in periphery type C (b, c, arrowheads). (a) *G. oocystiformis* Prescott strain 126, showing periphery type A (Supplementary Fig. 4). (b) *G. miyajii* Tos.Takah. & Nozaki sp. nov. strain Thu10, showing periphery type C (Supplementary Fig. 4). (c) *G. bhattacharyae* Tos.Takah. & Nozaki sp. nov. strain 118, showing periphery type C (Supplementary Fig. 4).

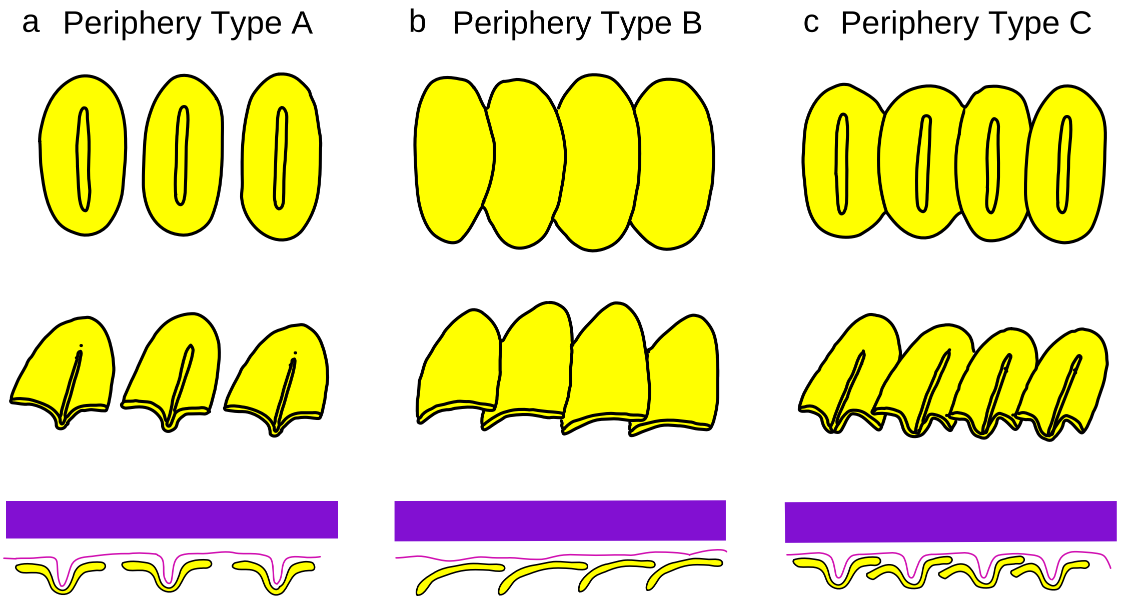

Supplementary Figure 4 | Diagrams of three types of the protoplast periphery in *Glaucocystis* species. Protoplast periphery beneath cell wall (purple) is composed of plasma membrane (magenta) and underlying flattened vesicles (yellow). Based on the present and previous studies[15,16]. (a) Periphery type A. Note that the flattened vesicles (yellow) are separated from one another and that the protoplast periphery exhibits numerous bar-like grooves and invaginations at intervals of 500–800 nm. (b) Periphery type B. Note that the flattened vesicles often overlap one another and that the protoplast periphery is almost smooth or flat, lacking grooves or invaginations. (c). Periphery type C. Note that the flattened vesicles often overlap one another and that the protoplast periphery exhibits numerous bar-like grooves and invaginations at intervals of 200–600 nm.

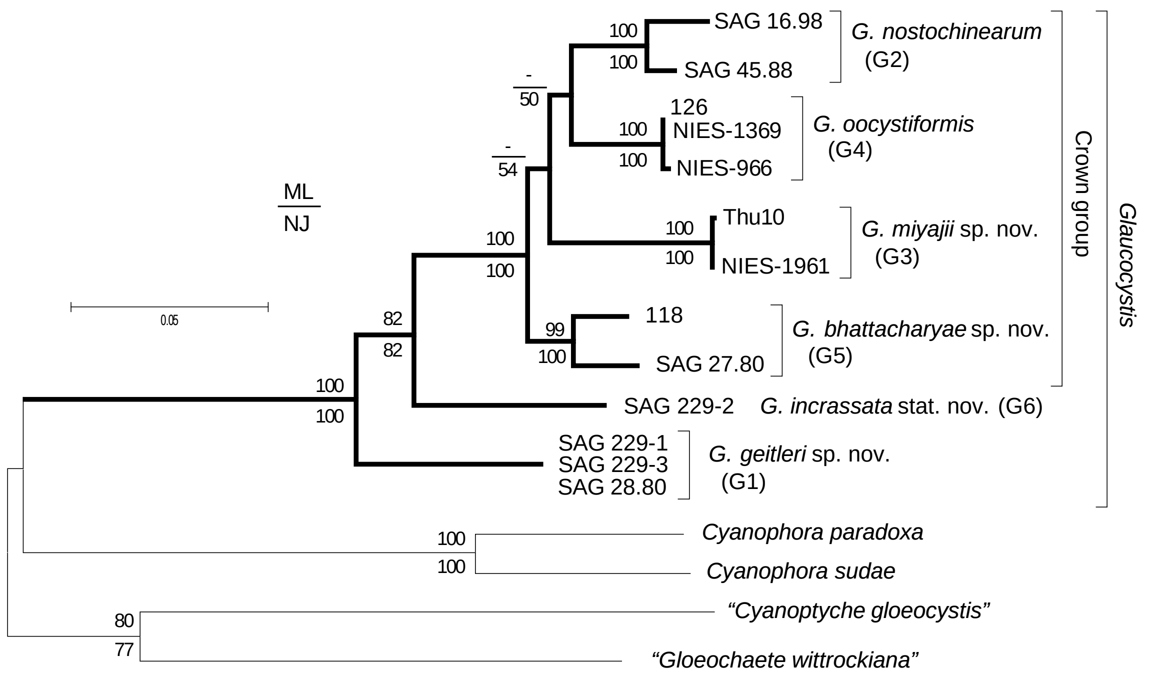

Supplementary Figure 5 | Maximum likelihood (ML) tree based on concatenated analyses of 1,461 and 750 base pairs of the coding regions of the plastid *psaB* and *psbA* genes, respectively, from 13 strains of six *Glaucocystis* species and four strains of other glaucophyte genera (Supplementary Table 1). Branch lengths are proportional to the genetic distances, which are indicated by the scale bar above the tree. Numbers above branches represent ≥50% bootstrap values (based on 1,000 replications) of the ML analyses and below branches are ≥50% bootstrap values (based on 1,000 replications) of neighbour-joining (NJ). For details of the phylogenetic methods, see Methods Section in the main text. G1–G6 within parentheses near the *Glaucocystis* species names represent the corresponding phylogenetic groups resolved previously[17].

a

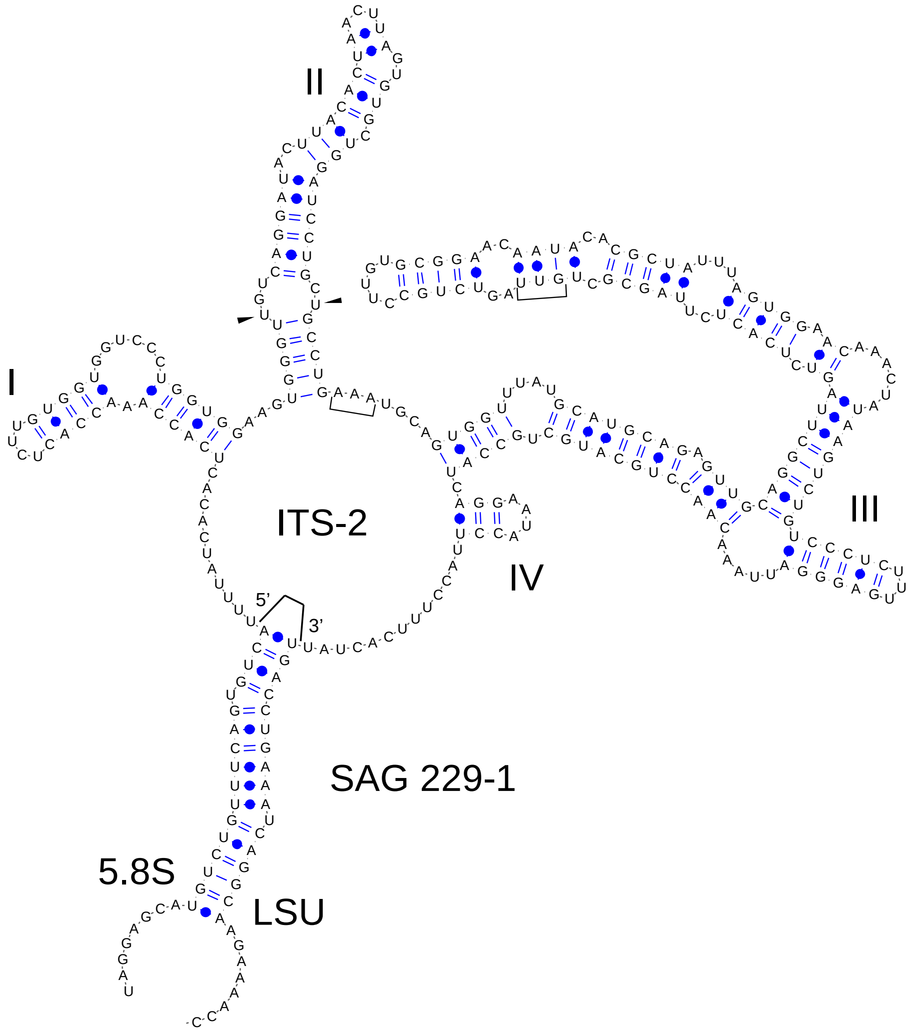

b

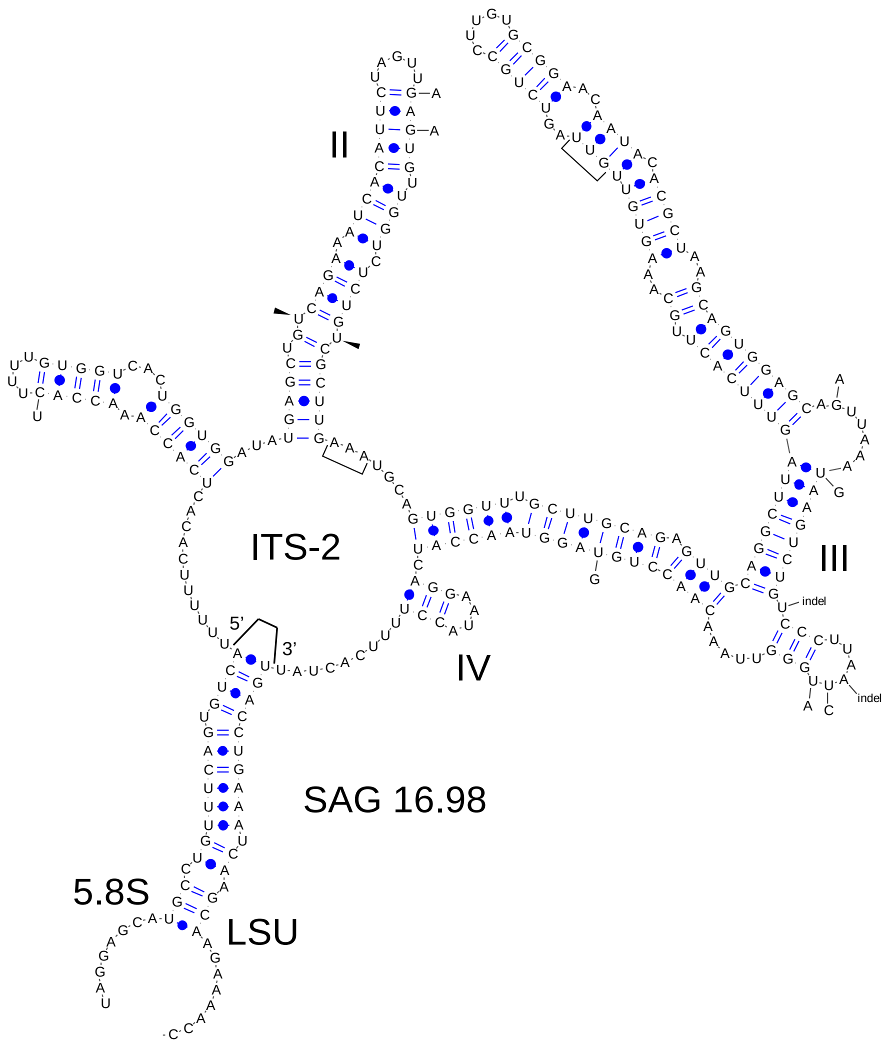

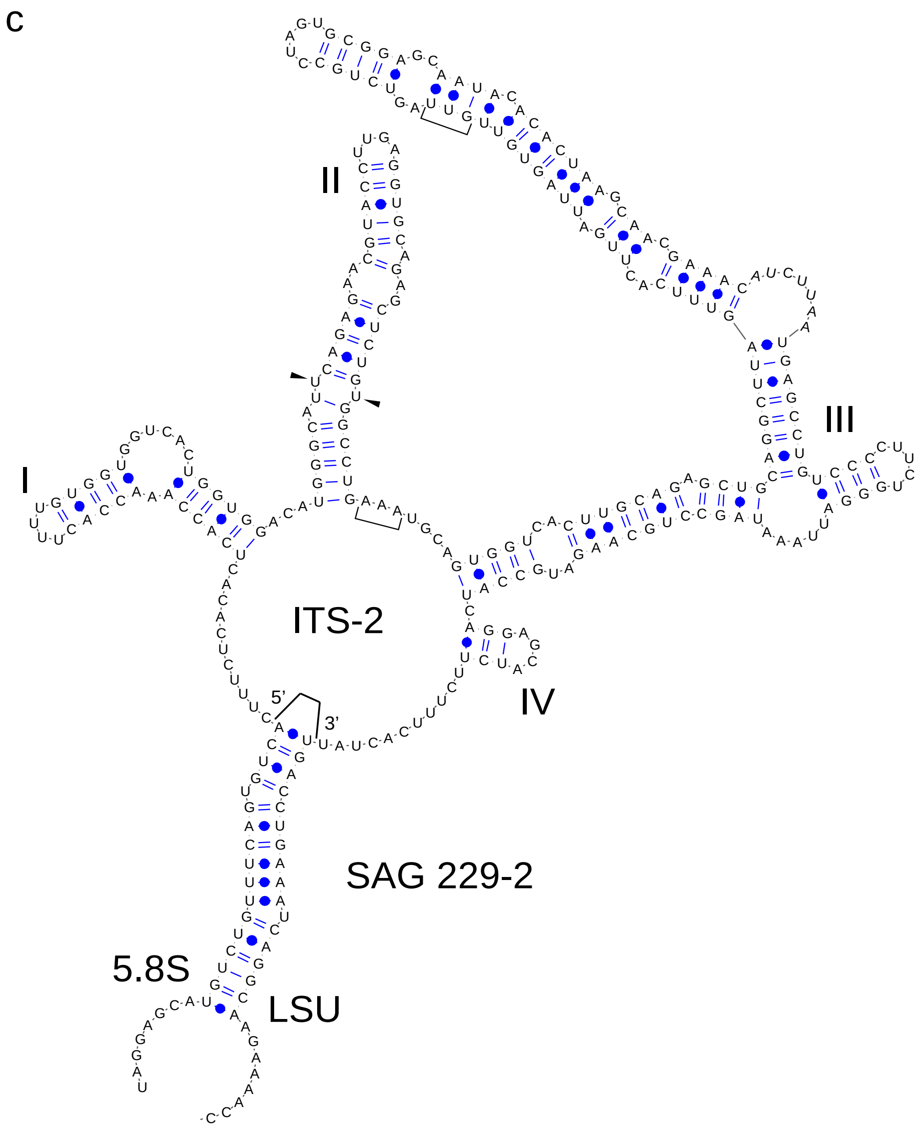
c
II
I
III
ITS-2
IV
5'
3'
SAG 229-2
5.8S
LSU

d

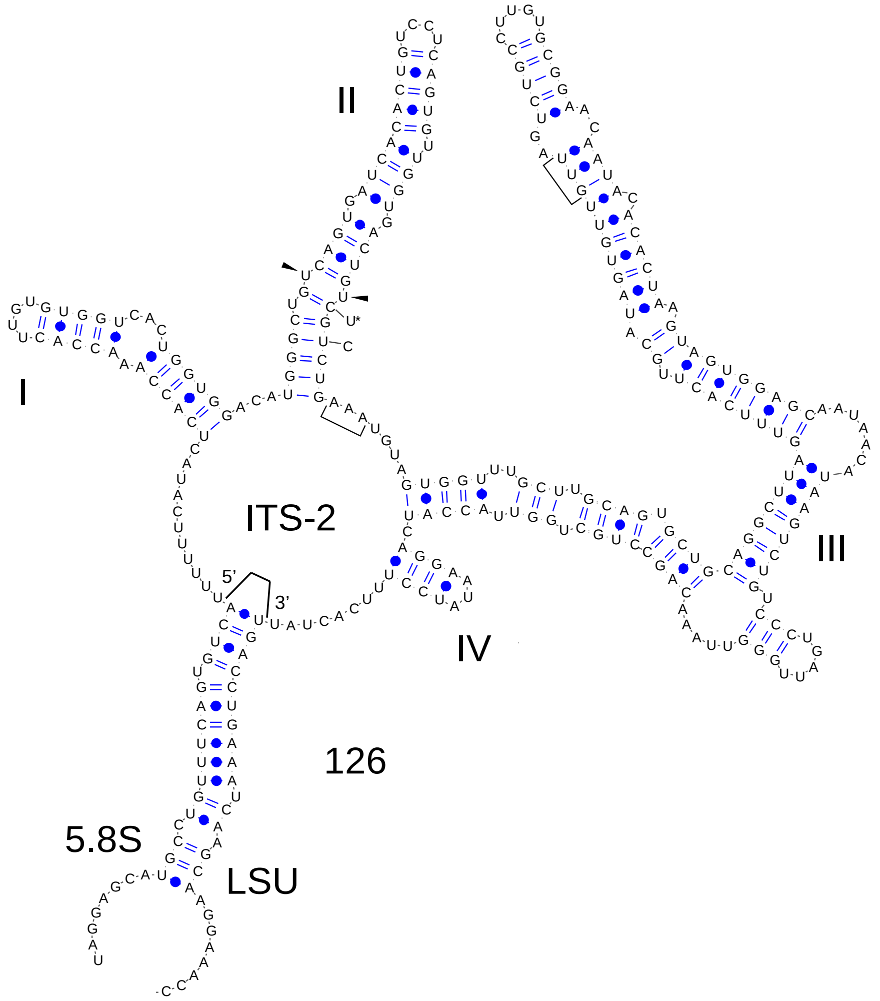

e

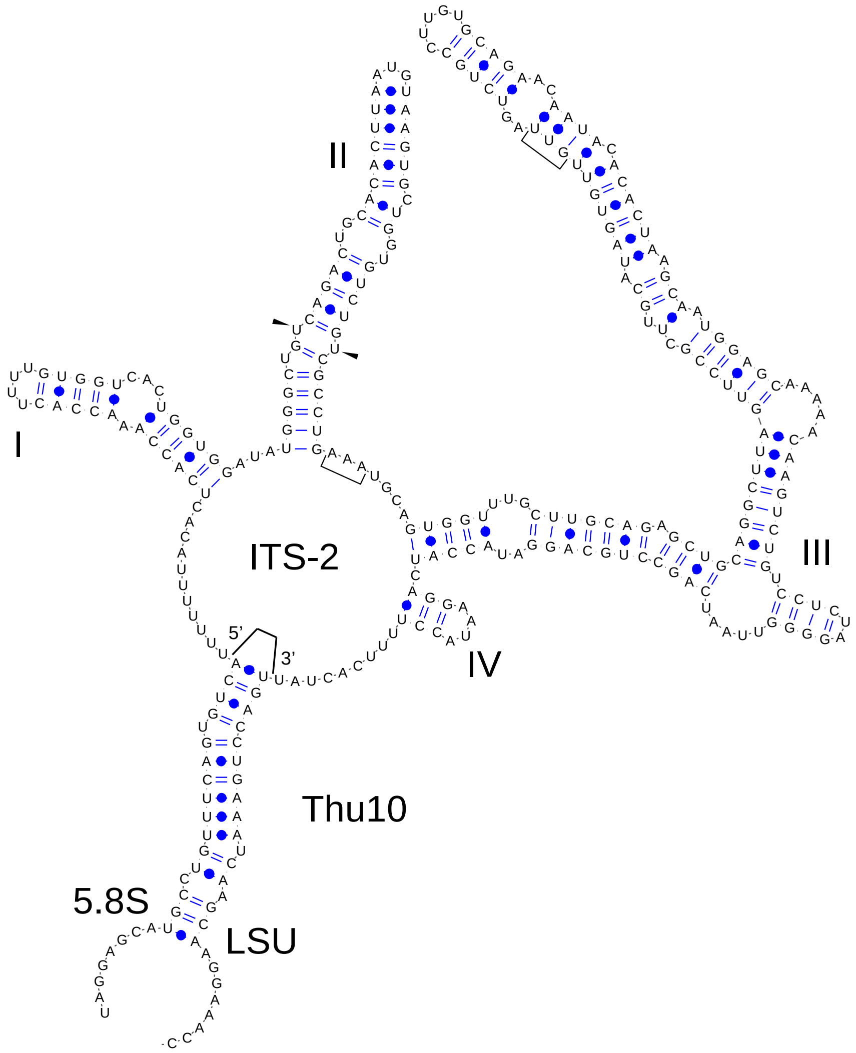

f

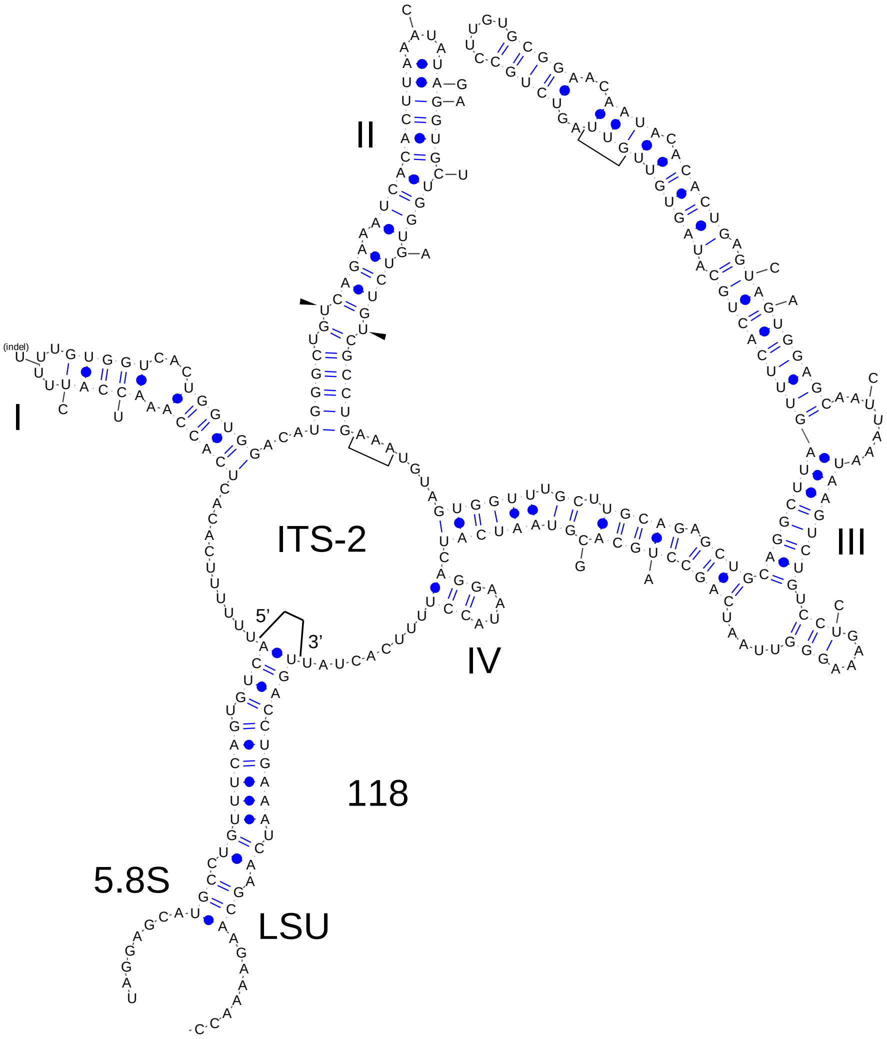

Supplementary Figure 6 | Secondary structures of nuclear *r*DNA ITS-2 of six species of *Glaucocystis*. Note that ITS-2 secondary structures were highly conserved within the genus *Glaucocystis* and that they had four helices with helix III as the longest, U-U mismatches (arrowheads) in the helix II with AAA motif (bracket) between the helix II and III and GYU motif (bracket) near the 5' site apex of helix III. (a) *G. geitleri* E.G.Pringsh. *ex* Tos.Takah. & Nozaki sp. nov. strains SAG 229-1, SAG 229-3 and SAG 28.80. (b) *G. nostochinearum* Itzigs. *ex* Rabenh. strain SAG 16.98 with structural variations found in strain SAG 45.88 indicated by outside lines. (c) *G. incrassata* (Lemmerm.) Tos.Takah. & Nozaki stat. nov. strain SAG 229-2. (d) *G. oocystiformis* Prescott strain 126. Note that structural variations found in *G. oocystiformis* strains NIES-966 and NIES-1369 are indicated by outside lines. Asterisk indicating structural variation only found in strain NIES-1369. (e) *G. miyajii* Tos.Takah. & Nozaki sp. nov. strains Thu10 and NIES-1961. Note that no structural variation was found between the two. (f) *G. bhattacharyae* Tos.Takah. & Nozaki sp. nov. strain 118 with structural variations found in strain SAG 27.80 indicated by outside lines.

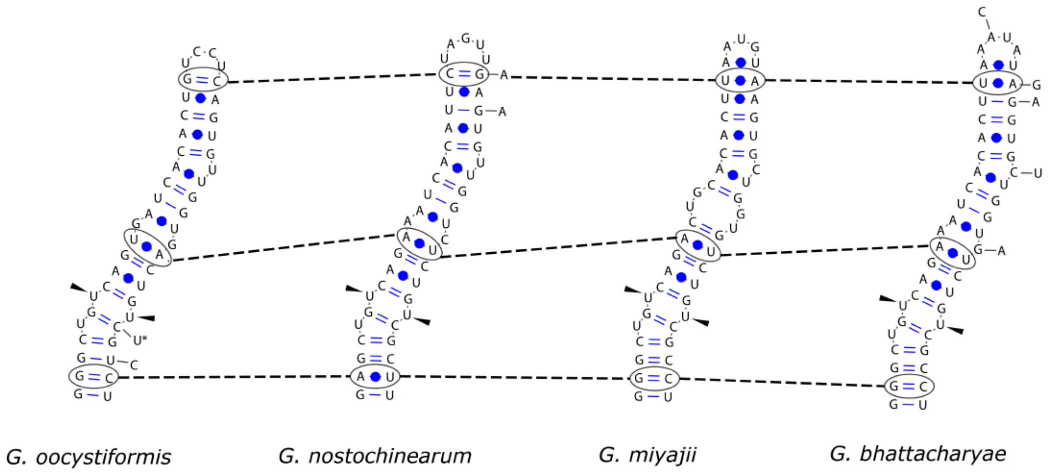

Supplementary Figure 7 | Comparison of the secondary structures of nuclear *r*DNA ITS-2 between species within crown group of *Glaucocystis* resolved in the present molecular phylogeny (Supplementary Fig. 5). Dotted lines indicate compensatory base changes between the helices. For complete secondary structures and structural variations indicated by outside lines, see Supplementary Fig. 6.

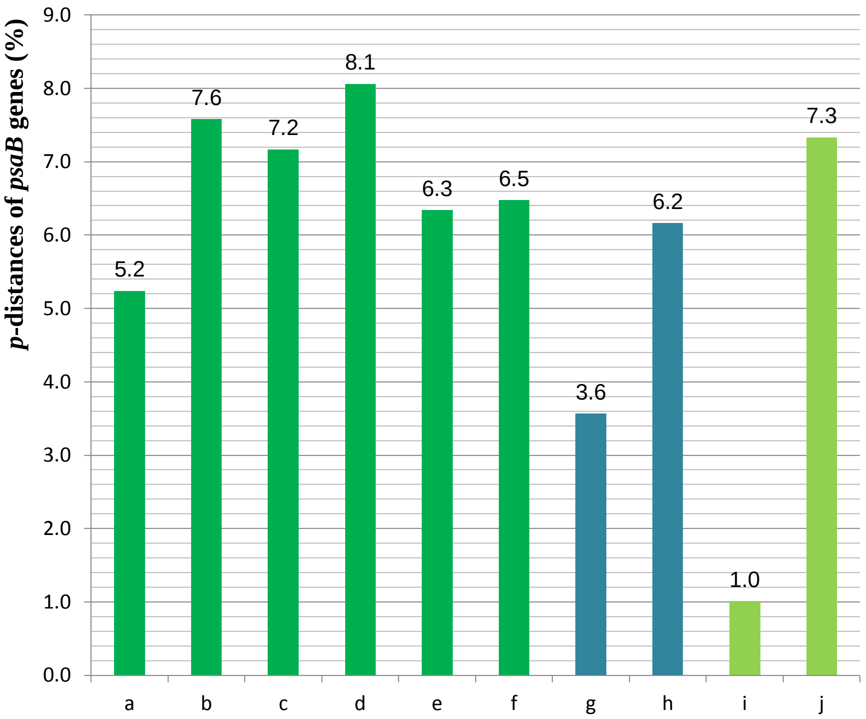

Supplementary Figure 8 | Nucleotide differences (%) or *p*-distances of *psaB* genes between *Glaucocystis* species within crown group resolved in the present molecular phylogeny (Supplementary Fig. 5) and *Cyanophora* and unicellular green algal genera[3]. The smallest difference is shown when multiple combinations of strains are present between sister species. (a) *G. oocystiformis* and *G. nostochinearum*, (b) *G. nostochinearum* and *G. miyajii* sp. nov., (c) *G. oocystiformis* and *G. miyajii* sp. nov., (d) *G. bhattacharyae* sp. nov. and *G. miyajii* sp. nov., (e) *G. bhattacharyae* sp. nov. and *G. oocystiformis*, (f) *G. bhattacharyae* sp. nov. and *G. nostochinearum*, (g) *Cyanophora cuspidata* and *C. kugrensii*, (h) *C. biloba* and *C. sudae*, (i) *Hafniomonas conica* and *H. turbinea* and (j) *Chlorogonium capillatum* and *Ch. euchlorum*.

Supplementary Note

Historical background

Members of the coccoid glaucophyte family Glaucocystaceae (Glaucocystales) are enclosed by a thick, cellulosic cell wall wherein they reproduce themselves[9,18]. This family includes two genera[9,18,19], *Glaucocystis* Itzigs. *ex* Rabenh. (1866)[13,14] and *Glaucocystopsis* Bourr. (1961)[20,21]; while *Glaucocystopsis* is monotypic and characterised by having just four plastids per cell, *Glaucocystis* has more than four plastids[9,20,21]. Early investigation of the genus *Glaucocystis* was performed using field-collected materials[22-26], establishing the genus concept. To date, seven species have been described within the genus, based on the cell wall characters or cell shape, as determined by LM[6-12,18,27]: *G. bullosa* (Kütz.)[4] Wille (1919)[5], *G. nostochinearum* Itzigs. *ex* Rabenh. (1866)[13,14], *G. cingulata* Bohlin (1897)[8], *G. duplex* Prescott (1944)[11,12], *G. oocystiformis* Prescott (1944)[11,12], *G. indica* R.J.Patel (1981)[6] and *G. reniformis* B.N.Prasad, R.K.Mehrotra & P.K.Misra (1984)[7] (Supplementary Table 2). No authentic strains (the culture used for the type specimen of the original description) have yet been established in any of the species. Although strains identified as the type species "*G. nostochinearum*" are available from culture collections worldwide, they lack morphological data for species identification[1,2] (Supplementary Table 1). No other species of *Glaucocystis* have been maintained in culture collections. Among the seven species, *G. nostochinearum* is considered a cosmopolitan species[10,12,28,29]. Coccoid glaucophyte algae were identified as *G. nostochinearum* from all over the world (Africa, Europe, Asia, Oceania, and North and South America)[12,28-32]. However, the species identifications were based only on LM findings[10,28], and different morphological characteristics of this species have been described depending on the authors or identifiers[9,12,28].

Pringsheim[33] identified an original isolate (SAG 229-2) as *G. nostochinearum* var. *incrassata* Lemmerm. (1908)[34]. He also provided a provisional name, *G. geitleri*

E.G.Pringsh. (1958) *nom. provis.*, for another original isolate (SAG 229-1)[33]. However, he did not compare these two strains with other strains identified as *G. nostochinearum*.

Differences in *Glaucocystis* as determined by light microscopy

Using light microscopy (LM), *Glaucocystis* strains 126, NIES-1369 and NIES-966 were clearly identified as *G. oocystiformis*[11,12] by their cell morphology (see below), even based on the traditional taxonomic system[9,11,12] (Supplementary Table 2). Other strains were identified as the type species *G. nostochinearum* using the traditional system (Supplementary Table 2), but we found LM-based differences that contributed to delineation of the five morphological species (Table 1).

The vegetative cells showed four distinct types that were diagnostic at the species level. These types could be distinguished from one another by differences in the shape of the cell wall at the cell poles (Table 1; Supplementary Fig. 1): 1) ellipsoidal cells with variable protrusions or nodules of the cell wall at the poles (*G. oocystiformis*), 2) barrel-shaped cells with cell wall thickening at the truncate poles [*G. incrassata* (Lemmerm.) Tos.Takah. & Nozaki stat. nov. and *G. geitleri*], 3) barrel-shaped cells without cell wall thickening at the truncate poles (*G. bhattacharyae* Tos.Takah. & Nozaki sp. nov.) and 4) ellipsoidal cells without distinctive cell wall characteristics at the poles (*G. nostochinearum* and *G. miyajii* Tos.Takah. & Nozaki sp. nov.). In addition, two types of mother cell walls were recognised. In all six *Glaucocystis* species, two, four or eight cells in the present study were surrounded by their mother cell wall to form a colony, but the degree of expansion of the mother cell wall differed among the species (Fig. 1). In *G. geitleri*, *G. nostochinearum*, *G. oocystiformis* and *G. miyajii*, the cells were arranged separately within the prominently expanded mother cell wall (Fig. 1a,b,d,e). In contrast, the mother cell wall of *G. incrassata* and *G. bhattacharyae* was not prominently expanded, such that the inside cells were arranged tightly as they were attached to one another (Fig. 1c,f). This difference in expansion of the mother cell wall corresponded to the ultrastructural differences in cellulose filaments of the mother cell

wall or colony surface under low accelerating voltages (LV) field-emission scanning electron microscopy (FE-SEM) (Fig. 2; Table 1; see also main text). Furthermore, colony cell number and cell size differed among these five species (Table 1).

Results of species evaluation based on the secondary structure of nuclear *r*DNA ITS-2 and genetic distances among *psaB* genes

As reported previously for species of *Cyanophora*[3], species of *Glaucocystis* delineated based on morphological differences (Table 1; Fig. 4) and phylogeny (Supplementary Fig. 5) were evaluated using the secondary structure of nuclear ribosomal DNA (*r*DNA) internal transcribed spacer (ITS)-2 (Supplementary Figs. 6, 7) and the genetic distances among the photosystem I P700 chlorophyll *a* apoprotein A2 (*psaB*) genes (Supplementary Fig. 8). Within each *Glaucocystis* species or the phylogenetic groups, no compensatory base change (CBC) was identified among the strains. Within the crown group (Supplementary Fig. 5), however, at least one CBC was detected between all pairs of species, except between *G. miyajii* and *G. bhattacharyae* (e.g. in helix II, Supplementary Fig. 7). Between the crown group species and *G. incrassata*, at least one CBC was also detected, whereas between the most basal *G. geitleri* and all other species, at least five CBCs were detected. Therefore, *Glaucocystis* species display sufficient genetic distance, based on the *psaB* gene, for separation into distinct species.

P-distances of *psaB* genes between species of *Glaucocystis* were calculated (Supplementary Fig. 8) within the crown group (Supplementary Fig. 5) and compared with the ranges exhibited between green algal and *Cyanophora* species[3]. Within the genus *Glaucocystis*, the crown group species *G. oocystiformis* and *G. nostochinearum* exhibited the smallest difference in *psaB* *p*-distance (5.2%), whereas the other *psaB* *p*-distances between each pair of species within the crown group ranged from 6.3% to 8.1% (Supplementary Fig. 8). These values fall within the ranges of the *psaB* *p*-distances (1.0–7.3%) seen between sister species in the unicellular green algae as well as the glaucophyte genus *Cyanophora* (Supplementary Fig. 8)[3]. Thus, compared with

several other genera, the six *Glaucocystis* species examined here display sufficient genetic distance for separation into distinct species.

Three *Glaucocystis* species found in a single field sample

Using new-generation electron microscopic (EM) methods, we discovered ultrastructural diversity capable of delineating six *Glaucocystis* species among various strains distributed globally. Since our novel strains established here based on a single field sample (Supplementary Table 1) were classified into three species in the present classification (as discussed in the main text), the fact that the taxon *G. nostochinearum* has been considered somewhat diverse or ambiguous[9,12,28] might be ascribable to the mixture of two or more *Glaucocystis* species in a single sample. Indeed, *G. nostochinearum* has been considered a cosmopolitan species[10,12,28], but the records may actually be based on several species that can be recognised here. The establishment, utilisation and maintenance of new clonal strains should enable further taxonomic progress through the application of molecular analyses and several microscopic methods. As discussed in the main text, in our novel taxonomic system (Table 1; Fig. 4), five species were recognised within the traditional species concept of the type species *G. nostochinearum* (Supplementary Table 2). Thus, we performed lectotypification and epitypification for the alga (see below) and described four new species (see *Taxonomic account* in the main paper).

Lectotypification of the original materials of *G. nostochinearum*

Syntype materials (Rabenhorst's exsiccata *Die Algen Europas* no. 1935[13,14]) are still present in several herbaria, including the Farlow Herbarium (FH), Harvard University, but exsiccated cells collected in 1864 cannot provide ultrastructural information (Supplementary Fig. 2a)[13,14]. The syntypic authentic strain was not established, and thus epitypification was required to establish a nomenclatural criterion for the alga. We obtained the submaterial of no. 1935 *G. nostochinearum* from the FH and prepared the

permanent slide R1935J (Supplementary Fig. 2a) for lectotypification, which is necessary for epitypification under the International Code of Nomenclature for algae, fungi and plants (ICN, formerly ICBN, International Code of Botanical Nomenclature) (see also *Taxonomic account* in the main paper). According to Rabenhorst[13,14], the cells of *G. nostochinearum* were ellipsoidal without truncate poles or thickening of the cell wall (Supplementary Fig. 2a). Strain SAG 16.98 has ellipsoidal vegetative cells and lacks such truncate poles and thickening (Supplementary Figs. 1, 2b,c). This strain originates from Germany (http://sagdb.uni-goettingen.de/)[1] (Supplementary Table 1), where the type locality of *G. nostochinearum* is located[13]. Thus, cells of SAG 16.98 were selected as the epitype material of *G. nostochinearum* (see the main text).

Supplementary References

1. Schlösser, U. G. SAG—Sammlung von Algenkulturen at the University of Göttingen. Catalogue of Strains 1994. *Bot. Acta* **107**, 113–186 (1994).

2. Kasai, F. *et al*. *NIES-Collection. List of Strains 8th Edition* (Japanese Journal of Phycology, Tsukuba, 2009).

3. Takahashi, T. *et al*. Five *Cyanophora* (Cyanophorales, Glaucophyta) species delineated based on morphological and molecular data. *J. Phycol.* **50**, 1058–1069 (2014).

4. Kützing, F. T. *Algarum Aquae Dulcis germanicum, Decas 16*, (Halle (Saale), 1836).

5. Wille, J. N. F. Algologische Notizen XXV-XXIX. XXIX.Studien in Agardh's Herbarium 8-15. 9. Über *Microcystis bullosa* (Kütz.) Menegh. und *M. gelatinosa* Menegh. (p.33-60). *Nyt Mag. f. Naturv.* **56**, 1–60 (1919).

6. Patel, R. J. *Glaucocystis indica* Patel sp. nov. from India. *Geophytology* **11**, 259–261 (1981).

7. Prasad, B. N., Mehrotra, R. K. & Misra, P. K. *Glaucocystis reniformis* sp. nov. from Andaman Islands. *Cryptogamie. Algologie* **5**, 79–84 (1984).

8. Bohlin, K. Die Algen der ersten Regnell'schen Expedition. I. Protococcoiden. *Bih. K. Svensk. Vet. -Akad. Handl.* **23**, 1–47 (1897).

9. Komárek, J. & Fott, B. *Chlorophyceae (Grünalgen); Ordnung: Chlorococcales* 446–555 (E. Schweizerbart'sche Verlagsbuchhandlung, Stuttgart, 1983).

10. Starmach, K. *Cyanophyta—Sinice. Glaucophyta—Glaukofity.* (Państwowe Wydawn. Naukowe, Warszawa, 1966).

11. Prescott, G. W. New species and varieties of Wisconsin algae. *Farlowia* **1**, 347–385 (1944).

12. Prescott, G. W. *Algae of the Western Great Lakes Area* (WC Brown Company, Dubuque, 1962).

13. Rabenhorst, L. G. *Die Algen Europas, Fortsetzung der Algen Sachsens, resp. Mittel-europas. Decades 94–95, Number 1935* (Dresden, 1866).

14. Rabenhorst, L. G. *Flora Europaea Algarum Aquae Dulcis et Submarinae. III. Algas Chlorophyllophyceas, Melanophyceas et Rhodophyceas Complectens* (Eduard Kummer, Leipzig, 1868).

15. Takahashi, T., Nishida, T., Saito, C., Yasuda, H. & Nozaki, H. Ultra-high voltage electron microscopy of primitive algae illuminates 3D ultrastructures of the first photosynthetic eukaryote. *Sci. Rep.* **5**, 14735 (2015).

16. Takahashi, T., Nishida, T., Saito, C., Yasuda, H. & Nozaki, H. A new type of 3D peripheral ultrastructure in *Glaucocystis* (Glaucocystales, Glaucophyta) as revealed by ultra-high voltage electron microscopy. *J. Phycol.* **52**, 486–490 (2016).

17. Chong, J., Jackson, C., Kim, J. I., Yoon, H. S. & Reyes-Prieto, A. Molecular markers from different genomic compartments reveal cryptic diversity within glaucophyte species. *Mol. Phylogenet. Evol.* **76**, 181–188 (2014).

18. Kies, L. & Kremer, B. P. Typification of the Glaucocystophyta. *Taxon* **35**, 128–133 (1986).

19. Skuja, H. L. *Allgemeiner Teil, Bakterien bis Gymnospermen* (eds Melchior, H. & Werdermann, E.) 56–57 (Gebr. Borntraeger, Berlin-Nikolassee, 1954).

20. Bourrelly, P. Un nouveau genre africain d'endocyanose: *Glaucocystopsis africana*: nov. gen. et nov. sp. *C. r. hebd. séances Acad. sci.* **251**, 416–418 (1960).

21. Bourrelly, P. Algues d'eau douce de la République de Côte d'Ivoire. *Bull. Inst. Franç. Afr. Noire, sér. A* **23**, 283–374 (1961).

22. Hieronymus, G. Beitrage zur Morphologie und Biologie der Algen. I. *Glaucocystis nostochinearum* Itzigsohn. *Beitr. Biol. Pfl.* **5**, 461–495 (1892).

23. Griffiths, B. M. On *Glaucocystis Nostochinearum*, Itzigsohn. *Ann. Bot.* **29**, 423–432 (1915).

24. Chodat, R. Sur un *Glaucocystis* et sa position systématique. *Bull. Soc. Bot. Genève* **2**, 42–49 (1919).

25. Geitler, L. Der Zellbau von *Glaucocystis Nostochinearum* und *Gloeochaete Wittrockiana* und die Chromatophoren-Symbiosetheorie von Mereschkowsky. *Arch. Protistenk.* **47**, 1–24 (1923).

26. Lagerheim, G. Ein nenes Beispiel des Vorkommens von Chromatophoren bei den Phycochromaceen. *Ber. dtsch. Bot. Ges.* **2**, 302–304 (1884).

27. Patel, R. J. & Isabella, G. Chlorococcales of Gujarat—India. *Vidya. B, Sci.* **22**, 1–9 (1979).

28. Prasad, B. N. *Glaucocystis nostochinearum* (Itzig.) Rabenhorst in India. *Bull. Bot. Soc.* **13**, 44–45 (1961).

29. Compère, P. Algues de la région du lac Tchad. V—Chlorophycophytes (1re partie) (1). *Cah. O. R. S. T. O. M. sér. Hydrobiol.* **10**, 77–118 (1976).

30. Gordon, D. P. *Ecosystem Services in New Zealand—Conditions and Trends* (ed Dymond, J. R.) 162–191 (Manaaki Whenua Press, Landcare Research, Lincoln(New Zealand), 2013).

31. de Azevedo Barros, C. F. Diversidade e ecologia do fitoplâncton em 18 lagoas naturais do médio Rio Doce. *Univ. Federal de Minas Gerais*, 1–119 (2010).

32. Whitton, B. A. *The Freshwater Algal Flora of the British Isles: An Identification Guide to Freshwater and Terrestrial Algae* (eds John, D. M., Whitton, B. A. & Brook, A. J.) 613 (Cambridge University Press, London, 2002).

33. Pringsheim, E. G. in *Studies in Plant Physiology* (ed Prat, S.) 165–184 (Czechoslovak Acad. Sci., Prague, 1958).

34. Lemmermann, E. Algologische Beiträge. *Arch. Hydrobiol. Planktonkde.* **4**, 165–192 (1908).

Supplementary Video 1 | Ultra-high voltage electron microscopic images and tomography of *Glaucocystis oocystiformis* Prescott strain 126 periphery. Corresponding to Fig. 3 in the main paper.

Supplementary Video 2 | Ultra-high voltage electron microscopic images and tomography of *Glaucocystis miyajii* Tos.Takah. & Nozaki sp. nov. strain 118 periphery. Corresponding to Fig. 3 in the main paper.

Supplementary Video 3 | Ultra-high voltage electron microscopic images and tomography of *Glaucocystis bhattacharyae* Tos.Takah. & Nozaki sp. nov. strain Thu10 periphery. Corresponding to Fig. 3 in the main paper.
